# Comparison of predictive models for estimating scorpion, snake, and spider envenomation under warming scenarios in São Paulo State, Brazil

**DOI:** 10.1007/s00484-026-03353-8

**Published:** 2026-11-03

**Authors:** Livia da Silva Freitas, Flavio Manoel Rodrigues da Silva Júnior

**Affiliations:** 1 Federal University of Rio Grande - FURG, Av. Itália, km 8, Campus Carreiros, Rio Grande, Rio Grande do Sul 96203-900 Brazil; 2 Federal University of Alagoas – UFAL, Av. Lourival Melo Mota, s/n, Tabuleiro do Martins, Maceió, Alagoas 57072-900 Brazil

**Keywords:** Venomous animals, Predictive modeling, Machine learning, Random forest, Temperature increase, Epidemiological surveillance

## Abstract

Venomous animal incidents represent an important public health issue and may be influenced by meteorological conditions as well as land use and land cover characteristics. However, it remains unclear which predictive approaches perform best in estimating these events and how increasing temperatures may alter their spatial distribution. This study compared different predictive models to estimate monthly incidence rates of scorpion, snake, and spider envenomation across the municipalities of São Paulo State, Brazil, from 2010 to 2024, and evaluated the potential impact of hypothetical warming scenarios (+ 1 °C, + 2 °C, and + 4 °C). Multiple linear regression, Generalized Additive Models (GAM), Extreme Gradient Boosting (XGBoost), and Random Forest models were compared using meteorological and land use/land cover variables. Random Forest achieved the best predictive performance for all three animal groups. Land use and land cover variables, particularly the proportion of urban area, were the most important predictors of the current spatial distribution of incidents. In contrast, temperature was the main factor associated with the projected changes under the simulated warming scenarios. Increasing temperatures resulted in higher predicted incidence rates for all animal groups, with the greatest increases observed for scorpion envenomation, revealing substantial spatial heterogeneity among municipalities. These findings indicate that the factors associated with the current distribution of venomous animal incidents are not necessarily the same as those driving their response to rising temperatures, highlighting the importance of integrating meteorological and land use and land cover variables to improve future projections and support public health surveillance and planning.

**Supplementary Information:**

The online version contains supplementary material available at https://doi.org/10.1007/s00484-026-03353-8.

## Introduction

Venomous animal incidents represent a major public health concern in tropical and subtropical regions. It is estimated that 4.5 to 5.4 million snakebites occur worldwide each year, resulting in approximately 1.8 to 2.7 million envenomation cases, 81,000 to 138,000 deaths, and hundreds of thousands of cases of permanent disability (Afroz et al. 2024; Chowdhury et al. 2024; WHO, 2019). Although snakebite envenoming is the most extensively studied component of this public health problem, incidents involving scorpions, spiders, and other venomous animals also contribute substantially to the global burden of morbidity, particularly in low- and middle-income countries. In Brazil, these incidents are subject to mandatory notification and have shown an increasing trend over recent decades, accompanied by marked spatial heterogeneity and population-level inequalities (Souza et al. 2022). In addition to their direct health impacts, these incidents impose substantial social and economic burdens on both healthcare systems and affected populations (da Silva Freitas et al. 2026).

The occurrence of venomous animal incidents is closely associated with the distribution, abundance, and activity of these organisms, which are influenced by environmental factors such as temperature, precipitation, humidity, and land use and land cover. Changes in these conditions can alter species behavior, reproduction, and geographic distribution, thereby affecting the risk of human exposure (Chowdhury et al. 2024; da Silva Freitas et al. 2024; Martín et al., 2021). In this context, climate change and land use and land cover change have been identified as key drivers of the future dynamics of venomous animal incidents. Processes such as agricultural expansion, habitat fragmentation, and urbanization can modify the interface between natural environments and human settlements, thereby increasing contact between people and venomous animals (Martín et al., 2021; Bravo-Vega et al. 2023).

In recent years, studies on venomous animal incidents, particularly snakebite envenomation, have increasingly employed spatial analyses, species distribution models, and machine learning algorithms to integrate epidemiological, environmental, and climatic data. These approaches have enabled the identification of high-risk areas, improved the understanding of spatiotemporal patterns, and supported projections of the potential impacts of climate change and land use change (Pintor et al. 2021; Yousefi et al. 2020; Yousefi et al., 2023; Chowdhury et al. 2024). However, the performance of these approaches may vary depending on data characteristics and the complexity of the underlying ecological relationships. Despite the growing number of studies, recent reviews have highlighted the diversity of methodologies employed and emphasized the need for comparative studies evaluating the performance of different predictive approaches (Gololo et al. 2025; Pintor et al. 2021). Furthermore, it remains unclear whether the environmental factors explaining the current distribution of venomous animal incidents are the same as those determining their sensitivity under future climate warming scenarios. This knowledge gap limits the identification of the most appropriate methods for future projections and reduces the ability to anticipate spatial changes in the risk of venomous animal incidents under ongoing environmental change.

São Paulo State is the most populous state in Brazil and provides a particularly relevant setting for investigating these questions due to its high population density and pronounced environmental heterogeneity, encompassing forest remnants, extensive agricultural areas, and highly urbanized landscapes. Accordingly, this study aimed to compare different predictive modeling approaches for estimating the monthly incidence rates of scorpion, snake, and spider envenomation across the municipalities of São Paulo State between 2010 and 2024, identify the meteorological and land use factors associated with these incidents, and, based on the best-performing model, project changes in the expected incidence rates under temperature increase scenarios.

## Methods

### Study design

This longitudinal ecological study was based on secondary data on scorpion, snake, and spider envenomation recorded across the 645 municipalities of São Paulo State, Brazil, between January 2010 and December 2024. Epidemiological, meteorological, population, and land use and land cover data were integrated to investigate the influence of environmental factors on incidence rates and to estimate their behavior under different temperature increase scenarios.

### Epidemiological and population data

Epidemiological data were obtained from the Brazilian Notifiable Diseases Information System (Sistema de Informação de Agravos de Notificação-SINAN), made available by the Department of Informatics of the Brazilian Unified Health System (Departamento de Informática do Sistema Único de Saúde-DATASUS). Notifications of scorpion, snake, and spider envenomation were included and aggregated by municipality, year, and month of occurrence. All analyses were conducted separately for each animal group.

Annual municipal population estimates were obtained from the Brazilian Institute of Geography and Statistics (Instituto Brasileiro de Geografia e Estatística-IBGE). Monthly incidence rates were calculated per 100,000 inhabitants using the number of incidents recorded in each municipality and the corresponding annual population estimate, which was assigned to all months within the respective year.

### Environmental variables

Meteorological variables were obtained from reanalysis data provided by the Copernicus Atmosphere Monitoring Service (CAMS) and included monthly mean air temperature (°C), monthly accumulated precipitation (mm), monthly mean relative humidity (%), and monthly mean wind speed (m/s). Data were processed at the municipal level on a monthly basis throughout the study period.

Land use and land cover data were obtained from Collection 9 of the MapBiomas Project for the period from 2010 to 2024. For each municipality and year, the proportion of the municipal territory occupied by each land use and land cover class was calculated as a fraction of the total municipal area. The original MapBiomas classes were grouped into five categories: forest, grasslands and wetlands, agriculture, urban area, and water. The correspondence between the original classes and the categories used in this study is presented in Supplementary Table 1.

### Predictive modelling

For each animal group, four predictive models were evaluated: multiple linear regression, Generalized Additive Models (GAM), Extreme Gradient Boosting (XGBoost), and Random Forest. The response variable was the monthly incidence rate per 100,000 inhabitants, and the predictor variables included temperature, precipitation, relative humidity, wind speed, and the municipal proportions of forest, grasslands and wetlands, agriculture, urban area, and water.

The dataset was randomly divided into training (80%) and testing (20%) sets. Predictive performance was assessed on the testing set using the Root Mean Squared Error (RMSE), Mean Absolute Error (MAE), and coefficient of determination (R^2^). The model with the lowest RMSE and MAE and the highest R^2^ was considered the best-performing model.

Random Forest models were fitted using the *ranger* package in R with 500 decision trees. Predictor importance was assessed using *permutation importance*, calculated as the increase in *out-of-bag* prediction error after randomly permuting the values of each predictor variable. Higher values indicate a greater contribution of the corresponding variable to the predictive performance of the model.

### Warming scenarios

To evaluate the potential effects of climate warming, hypothetical scenarios of increases in monthly mean temperature of + 1 °C, + 2 °C, and + 4 °C were simulated. These increments were selected because they represent warming magnitudes widely discussed in reports of the Intergovernmental Panel on Climate Change (IPCC) and are frequently used in climate impact studies. The + 1 °C scenario represents moderate warming consistent with recent trends observed in many regions worldwide, whereas the + 2 °C scenario corresponds to a key global warming threshold widely adopted in climate change impact assessments. The + 4 °C scenario was included to represent an extreme warming condition associated with high greenhouse gas emission pathways projected for the twenty-first century (IPCC, 2023).

The simulated scenarios do not represent complete climate projections but rather sensitivity analyses designed to isolate the potential effect of increasing temperature on the predicted incidence rates while keeping all other environmental variables constant. Projected changes were calculated as the difference between the predicted incidence rate under each warming scenario and the predicted incidence rate under current conditions. For the + 4 °C scenario, this difference was defined as the incidence rate change (Δ incidence rate = predicted incidence rate under the + 4 °C scenario − predicted incidence rate under current conditions). This indicator was aggregated at the municipal level to estimate the projected mean change for each animal group.

### Spatial analyses

The projected mean municipal change under the + 4 °C scenario was mapped using the municipal boundaries provided by the Brazilian Institute of Geography and Statistics (IBGE) in the SIRGAS 2000 coordinate reference system (EPSG:4674). Maps were produced using a common color scale for all three animal groups to facilitate comparison of the magnitude of the projected changes. Municipalities with no recorded incidents for a given animal group were not assigned specific estimates.

As an exploratory analysis, Pearson correlation coefficients were calculated between the mean municipal incidence rate change under the + 4 °C scenario and the mean environmental characteristics of each municipality, including temperature, precipitation, relative humidity, forest cover, agricultural land, and urban area. For each correlation, the correlation coefficient (*r*), *p* value, and corresponding 95% confidence interval (95% CI) were estimated. Municipalities with the largest projected increases and the largest reductions (or smallest increases) in predicted incidence rates under the + 4 °C scenario were also identified.

All analyses were performed in R (R Core Team, 2026) using the *ranger* package for Random Forest modelling, *dplyr* for data manipulation, *sf* for spatial processing, and *ggplot2* for data visualization.

## Results

### Predictive model performance

Random Forest achieved the best predictive performance for all three animal groups, with the lowest RMSE and MAE values and the highest R^2^ compared with multiple linear regression, Generalized Additive Models (GAM), and Extreme Gradient Boosting (XGBoost) (Table 1). This superior performance suggests that the relationships between environmental variables and incidence rates include nonlinear components that were not fully captured by the linear models.

**Table 1:** Comparison of the predictive performance of multiple linear regression, generalized additive models (GAM), extreme gradient boosting (XGBoost), and random forest for scorpion, snake, and spider envenomation

Animal	Model	RMSE	MAE	*R* ^2^
Scorpions	Linear	31.59	21.52	0.173
	GAM	29.54	19.56	0.277
	XGBoost	25.21	16.26	0.473
	Random Forest	22.03	13.27	0.598
Snakes	Linear	10.31	6.74	0.105
	GAM	8.99	5.43	0.32
	XGBoost	7.02	3.64	0.584
	Random Forest	6.11	2.89	0.686
Spiders	Linear	10.63	7.03	0.111
	GAM	9.39	5.78	0.308
	XGBoost	7.53	4.25	0.554
	Random Forest	6.57	3.46	0.66

For scorpion envenomation, Random Forest achieved an RMSE of 22.03, an MAE of 13.27, and an R^2^ of 0.598. Model performance was even higher for snake envenomation, with an RMSE of 6.11, an MAE of 2.89, and an R^2^ of 0.686. For spider envenomation, Random Forest also outperformed the other models, achieving an RMSE of 6.57, an MAE of 3.46, and an R^2^ of 0.660.

### Variable importance in the random forest models

The importance of predictor variables was assessed using permutation importance (Table 2). Across all models, urban area was the most influential predictor, followed primarily by agricultural land and forest cover.

**Table 2:** Permutation importance of environmental predictor variables in the random forest models for scorpion, snake, and spider envenomation

Animal	Variable	Raw permutation importance
Scorpions	Urban area	910.76
	Agricultural area	815.17
	Forest cover	713.31
	Water	550.57
	Wetlands and grasslands	442.55
	Humidity	108.4
	Precipitation	107.66
	Temperature	102.53
	Wind speed	50.3
Snakes	Urban area	170.92
	Agricultural area	105.35
	Forest cover	90.88
	Water	59.34
	Wetlands and grasslands	49.47
	Humidity	13.64
	Temperature	6.28
	Wind speed	3.59
	Precipitation	3.52
Spiders	Urban area	151.69
	Agricultural area	102.1
	Forest cover	102.06
	Wetlands and grasslands	61.7
	Water	51.86
	Humidity	12.39
	Temperature	9.21
	Wind speed	5.03
	Precipitation	4.46

Values represent the raw permutation importance scores, calculated as the increase in out-of-bag prediction error after randomly permuting each predictor variable. Higher values indicate a greater contribution of the predictor to model performance. These scores should be interpreted only in relative terms within each model and are not directly comparable across animal groups

For scorpion envenomation, the most important predictors were urban area, agricultural land, forest cover, water, and grasslands and wetlands. Meteorological variables showed lower relative importance, with similar contributions from relative humidity, precipitation, and temperature, whereas wind speed had the lowest importance. For snake envenomation, urban area was also the most influential predictor, followed by agricultural land, forest cover, water, and grasslands and wetlands. A similar pattern was observed for spider envenomation, for which urban area, agricultural land, and forest cover accounted for the highest predictor importance.

### Temporal performance of the random forest model

**Fig. 1:**
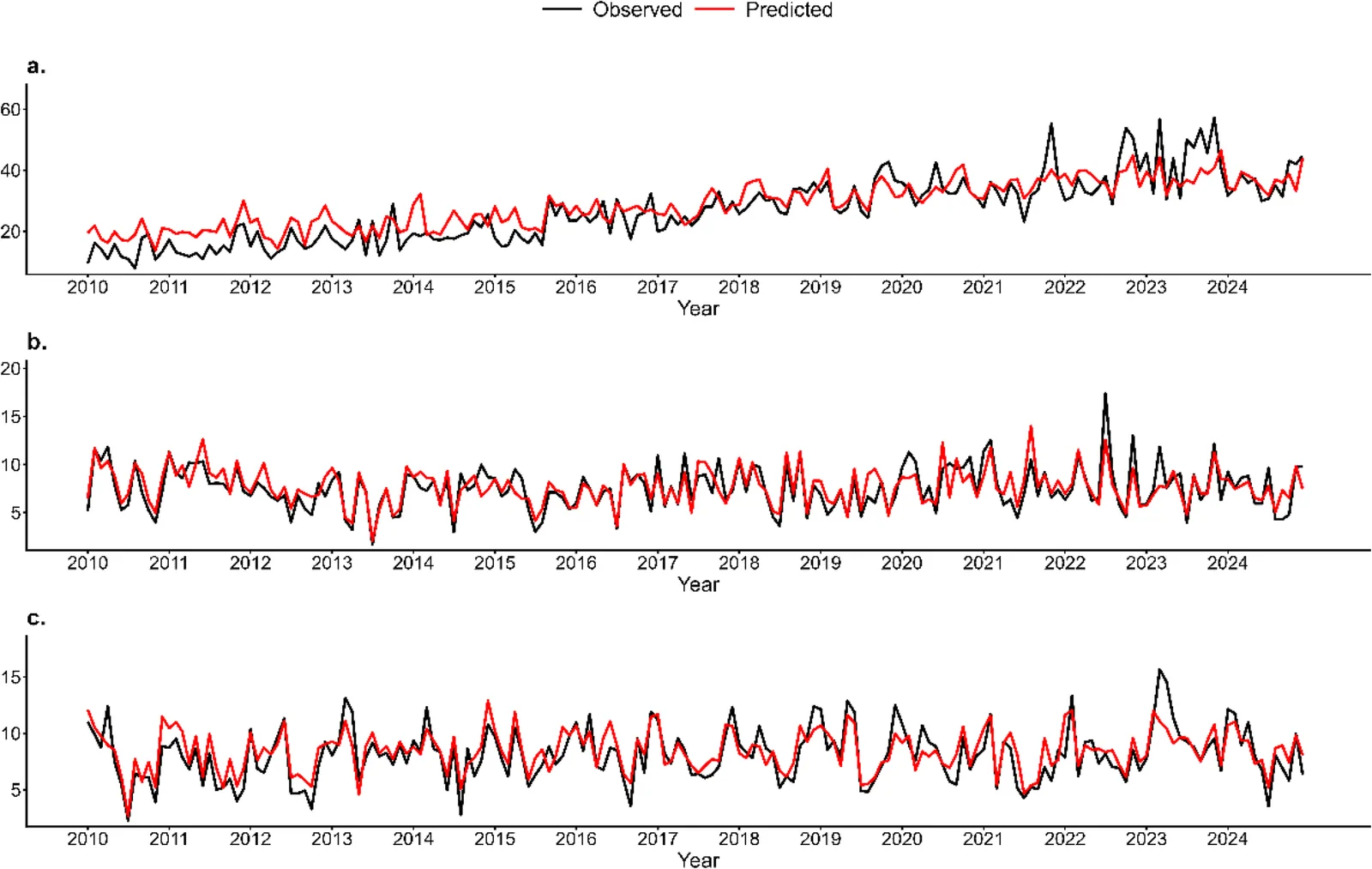
Observed and predicted monthly incidence rates of (**a**) scorpion, (**b**) snake, and (**c**) spider envenomation in São Paulo State, Brazil, from 2010 to 2024, estimated using the Random Forest model

Comparison between observed and predicted monthly incidence rates showed that the Random Forest model was able to reproduce the overall temporal trends for all three animal groups (Fig. 1). For scorpion envenomation, the model successfully captured the increasing trend over the study period, although it smoothed some of the observed peaks, particularly in the most recent years (Fig. 1a). For snake (Fig. 1b) and spider envenomation (Fig. 1c), the observed and predicted time series showed a greater degree of overlap, indicating good model performance in reproducing the average monthly variation of these groups.

Overall, the visual assessment of temporal model performance supports the suitability of Random Forest as the primary model for the subsequent warming scenario analyses. The tendency to smooth extreme values, particularly for scorpion envenomation, suggests that the model adequately captured the overall temporal pattern while showing a lower ability to reproduce the most pronounced peaks.

### Warming scenarios

Projections under the warming scenarios indicated progressive increases in the predicted mean incidence rates for all three animal groups, with the largest increase observed for scorpion envenomation (Table 3). Under current conditions, the predicted mean incidence rate for scorpion envenomation was 30.53 cases per 100,000 inhabitants. This rate increased to 31.87 under the + 1 °C scenario, 33.53 under the + 2 °C scenario, and 36.86 under the + 4 °C scenario, corresponding to an absolute increase of 6.33 cases per 100,000 inhabitants and a relative increase of 20.8% under the most extreme warming scenario.

**Table 3:** Predicted mean incidence rates, absolute changes (Δ incidence rate), and percentage increases under hypothetical warming scenarios (+ 1 °C, + 2 °C, and + 4 °C)

Animal group	Scenario	Predicted mean incidence rate (per 100,000 inhabitants)	Δ Incidence rate (per 100,000 inhabitants)	Percentage increase (%)
Scorpions	Current	30.53	0.0	0
	+ 1 °C	31.87	1.34	4.4
	+ 2 °C	33.53	3.00	9.9
	+ 4 °C	36.86	6.33	20.8
Snakes	Current	7.95	0.0	0
	+ 1 °C	8.13	0.18	2.2
	+ 2 °C	8.38	0.43	5.4
	+ 4 °C	8.81	0.86	10.8
Spiders	Current	8.57	0.00	0
	+ 1 °C	8.81	0.24	2.9
	+ 2 °C	9.11	0.54	6.3
	+ 4 °C	9.63	1.06	12.4

For snake envenomation, the predicted mean incidence rate increased from 7.95 cases per 100,000 inhabitants under current conditions to 8.13, 8.38, and 8.81 under the + 1 °C, + 2 °C, and + 4 °C scenarios, respectively. This corresponded to an absolute increase of 0.86 cases per 100,000 inhabitants and a relative increase of 10.8%. Similarly, for spider envenomation, the predicted mean incidence rate increased from 8.57 to 8.81, 9.11, and 9.63 cases per 100,000 inhabitants under the + 1 °C, + 2 °C, and + 4 °C scenarios, respectively, corresponding to an absolute increase of 1.06 cases per 100,000 inhabitants and a relative increase of 12.4%.

Although all three animal groups showed increasing predicted incidence rates under the warming scenarios, the magnitude of the response differed among them. Under the + 4 °C scenario, scorpion envenomation exhibited the largest absolute and relative increase, with an increase of 6.33 cases per 100,000 inhabitants (20.8%), whereas the corresponding increases for snake and spider envenomation were 0.86 cases (10.8%) and 1.06 cases per 100,000 inhabitants (12.4%), respectively. These findings indicate greater sensitivity of scorpion envenomation to the simulated temperature increases.

### Spatial variation in the projected impact under the + 4 °C scenario

Under the + 4 °C warming scenario, the projected municipal incidence rate changes were more pronounced for scorpion envenomation, whereas snake and spider envenomation showed more moderate responses. For scorpion envenomation, the projected incidence rate changes ranged from − 18.48 to + 27.98 per 100,000 inhabitants, with a median of + 5.84 and a mean of + 6.59. For snake envenomation, projected changes ranged from − 13.21 to + 10.49 per 100,000 inhabitants, with a median of + 0.59 and a mean of + 0.87. For spider envenomation, projected changes ranged from − 9.10 to + 8.00 per 100,000 inhabitants, with a median of + 0.93 and a mean of + 1.29.

**Fig. 2:**
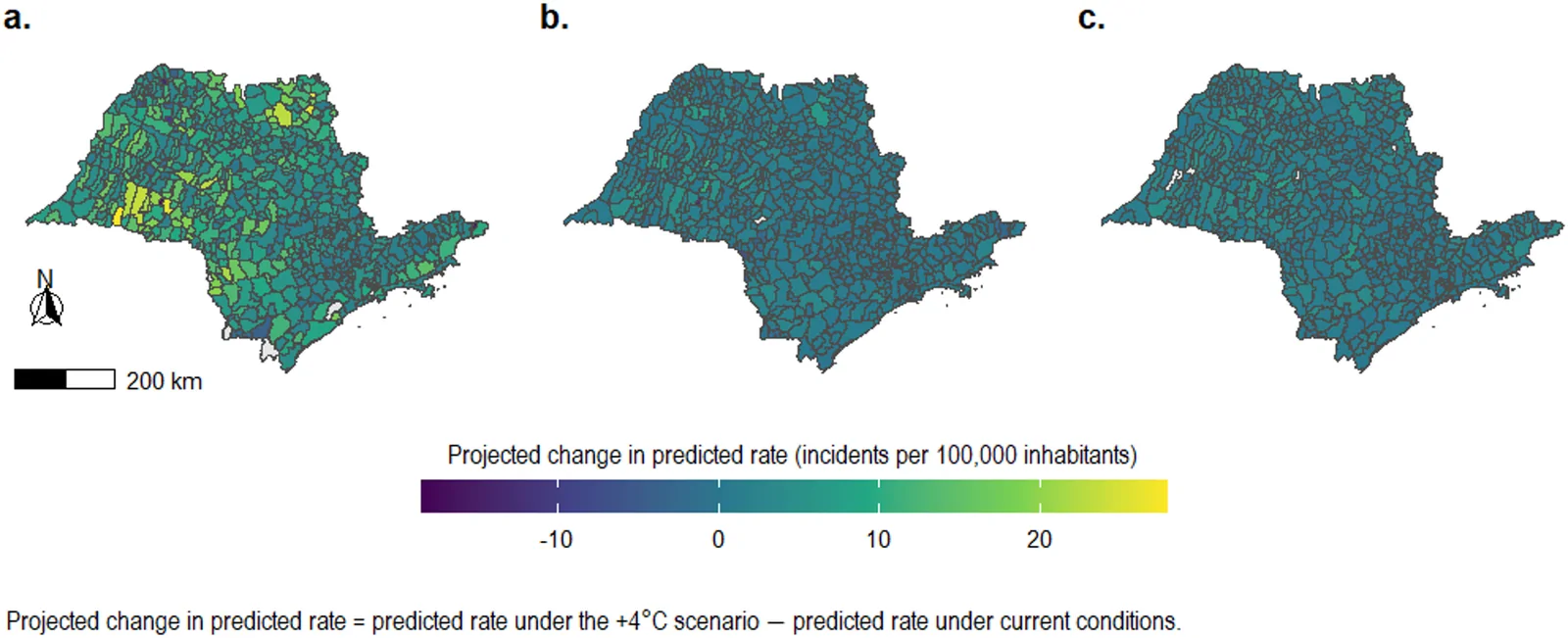
Spatial distribution of the projected change in predicted rates under the + 4 °C warming scenario for (**a**) scorpions, (**b**) snakes, and (**c**) spiders in São Paulo State, Brazil. The projected change was calculated as the predicted rate under the + 4 °C scenario minus the predicted rate under current conditions. Positive values indicate an increase in the predicted rate, whereas negative values indicate a decrease or lower sensitivity to the warming scenario

The spatial distribution of the projected incidence rate changes showed that the effects of warming were not homogeneous across São Paulo State (Fig. 2). Although the median change was positive for all three animal groups, indicating an overall tendency toward increased predicted incidence rates, the occurrence of negative values indicates that some municipalities experienced projected reductions or lower sensitivity under the + 4 °C scenario. This pattern suggests that the response to warming depends on local environmental and territorial characteristics. Furthermore, the presence of negative values indicates that the effect of temperature was not uniformly positive across the state, suggesting nonflinear relationships between temperature and predicted incidence rates.

The municipal rankings further reinforced this spatial heterogeneity. The largest projected increases were concentrated predominantly in municipalities located in the interior of São Paulo State. In contrast, municipalities showing projected reductions or lower sensitivity were more heterogeneously distributed, suggesting that the response to the simulated temperature increases depends on local combinations of climatic conditions, land use and land cover, and territorial characteristics. The complete ranking of municipalities with the largest projected increases and the largest projected reductions (or smallest increases) for each animal group is presented in Supplementary Table 2.

### Correlations between projected incidence rate changes under the + 4 °C scenario and municipal environmental characteristics

Correlations between the mean municipal incidence rate change under the + 4 °C scenario and environmental variables showed similar patterns across the three animal groups (Table 4). Temperature was positively correlated with the projected incidence rate change in all groups, with the strongest correlation observed for snake envenomation (*r* = 0.301), followed by scorpion (*r* = 0.238) and spider envenomation (*r* = 0.199). The proportion of agricultural land was also positively correlated with the projected increase across all three groups. In contrast, relative humidity, forest cover, and urban area were negatively correlated with the projected incidence rate change, indicating that municipalities characterized by higher humidity, greater forest cover, or greater urbanization tended to exhibit smaller projected increases under the + 4 °C scenario.

**Table 4:** Pearson correlation coefficients (r), 95% confidence intervals (95% CI), and p values for the associations between projected municipal incidence rate changes under the + 4 °C scenario and environmental characteristics

Animal	Variable	*r*	95% CI	*P* value
Scorpions	Temperature	0.238	(0.163; 0.310)	< 0.001
	Agricultural area	0.209	(0.133; 0.282)	< 0.001
	Humidity	-0.183	(-0.257; -0.107)	< 0.001
	Forest cover	-0.175	(-0.249; -0.098)	< 0.001
	Urban area	-0.144	(-0.219; -0.067)	< 0.001
	Precipitation	-0.096	(-0.172; -0.019)	0.015
Snakes	Temperature	0.301	(0.229; 0.370)	< 0.001
	Agricultural area	0.162	(0.085; 0.236)	< 0.001
	Humidity	-0.146	(-0.220; -0.069)	< 0.001
	Forest cover	-0.125	(-0.200; -0.048)	0.002
	Urban area	-0.080	(-0.157; -0.003)	0.041
	Precipitation	-0.017	(-0.094; 0.060)	0.662
Spiders	Temperature	0.199	(0.123; 0.272)	< 0.001
	Urban area	-0.176	(-0.250; -0.100)	< 0.001
	Agricultural area	0.166	(0.090; 0.241)	< 0.001
	Humidity	-0.095	(-0.172; -0.018)	0.016
	Forest cover	-0.093	(-0.169; -0.015)	0.019
	Precipitation	0.023	(-0.055; 0.101)	0.560

For scorpion envenomation, the largest projected increases were positively associated with temperature and agricultural land and negatively associated with relative humidity, forest cover, urban area, and precipitation. For snake envenomation, temperature showed the strongest positive correlation with the projected incidence rate change, whereas relative humidity, forest cover, and urban area were negatively associated; precipitation was not significantly associated with the projected change. For spider envenomation, the projected incidence rate change was also positively correlated with temperature and agricultural land and negatively correlated with urban area, relative humidity, and forest cover, whereas precipitation showed no significant association.

Taken together, these findings indicate that the factors associated with the current distribution of venomous animal incidents are not necessarily the same as those associated with their response to climate warming. Although land use and land cover variables were the main predictors of current incidence rates (Table 2), projected responses to warming were most strongly associated with temperature and, to a lesser extent, with agricultural land, lower relative humidity, and lower forest cover. These findings suggest that the determinants of current risk differ, at least in part, from those associated with future vulnerability under climate warming scenarios.

## Discussion

This study compared different predictive modelling approaches to estimate the monthly incidence rates of scorpion, snake, and spider envenomation across the municipalities of São Paulo State and evaluated the potential impact of increasing temperatures on these incidents. The results showed that models capable of capturing nonlinear relationships, particularly Random Forest, achieved superior predictive performance for all three animal groups. The main finding, however, was that the factors determining the current spatial distribution of venomous animal incidents are not necessarily the same as those explaining their response to increasing temperatures. Whereas land use and land cover variables, particularly the proportion of urban area, were the most important predictors of current incidence patterns, temperature was the primary factor associated with the projected changes under the warming scenarios.

The superior performance of Random Forest compared with multiple linear regression, Generalized Additive Models (GAM), and Extreme Gradient Boosting (XGBoost) suggests that the relationships between climatic, environmental, and territorial variables and the occurrence of venomous animal incidents are predominantly nonlinear and involve complex interactions among multiple factors (Fox et al. 2020; Nduwayezu et al. 2023). This behavior is expected in complex ecological and epidemiological systems, where tree-based algorithms generally outperform conventional statistical models, particularly when interactions among predictors, nonlinear responses, and spatial dependence are present (Schratz et al. 2019; Valavi et al. 2022; Keshavamurthy et al. 2022). In the context of venomous animal incidents, however, a wide range of modelling strategies has been employed, including regression models, spatiotemporal models, species distribution models, and machine learning algorithms, reflecting the diversity of research objectives and spatial scales adopted (Pintor et al. 2021). Despite these advances, recent reviews have highlighted the scarcity of studies directly comparing the performance of these approaches using epidemiological data (Gololo et al. 2025). In the present study, the superiority of Random Forest was particularly evident for scorpion envenomation, suggesting that linear approaches may underestimate the complexity of the mechanisms governing the spatiotemporal distribution of these incidents.

The high importance of land use and land cover variables indicates that landscape structure is one of the main spatial determinants of venomous animal incidents in São Paulo State. This association is consistent with the hypothesis that the risk of these incidents results from the interaction between ecological and human factors, with land use and land cover acting as a key driver of the frequency of contact between people and venomous animals (Martín et al., 2021). Consequently, anthropogenic landscape transformations play a central role in shaping current risk patterns, consistent with previous studies reporting associations between urbanization and increased incidence of venomous animal incidents, particularly those involving scorpions and spiders (Almeida-Gomes and Guariento 2025; Zanetta et al. 2020). For scorpion envenomation, this association is biologically plausible, as species such as *Tityus serrulatus* exhibit synanthropic behavior, high ecological plasticity, and a remarkable ability to colonize urban environments (Guerra-Duarte et al. 2023).

Urbanization increases the availability of shelters, food resources, and suitable microhabitats, thereby promoting scorpion proliferation and increasing human exposure (Amado et al. 2021; Brites-Neto et al. 2023). Higher temperatures associated with urban environments have also been linked to an increased risk of scorpion envenomation in São Paulo State (Chiaravalloti-Neto et al. 2023). However, recent studies indicate that the effects of urbanization are not uniform and may vary according to factors such as urban infrastructure, sanitation, climatic conditions, and the quality of surveillance systems (Duarte et al. 2024). These findings suggest that the relationship between urbanization and venomous animal incidents results from the interaction among ecological processes, landscape characteristics, and factors related to epidemiological surveillance.

Although land use and land cover were fundamental for explaining the current spatial distribution of venomous animal incidents, temperature was the factor most strongly associated with the projected changes under the warming scenarios. This finding is consistent with the central role of temperature in the ecology of ectothermic organisms, influencing their metabolism, activity, reproduction, behavior, and geographic distribution (da Silva Freitas et al. 2024).

Species distribution models for medically important species consistently identify thermal variables as key determinants of environmental suitability, driving shifts in the geographic distribution of suitable habitats under climate change scenarios (Hosseinzadeh 2020, Hosseinzadeh 2025; Martín et al., 2021; Yousefi et al. 2020). Complementing these findings, studies integrating ecological and epidemiological data indicate that such shifts may alter the spatial distribution of envenomation risk and improve the identification of areas that are likely to become more vulnerable under future climate conditions (Yousefi et al., 2023; Chowdhury et al. 2024).

This distinction has important implications for public health surveillance. Recent evidence indicates that increasing temperatures may alter the risk of venomous animal incidents and reinforces the need to incorporate climate information into surveillance and prevention strategies (da Silva Freitas et al. 2025; Martín et al., 2021). Consequently, municipalities that currently report low incidence rates may become more vulnerable if they are located in areas that are environmentally more sensitive to increasing temperatures, making surveillance and planning based solely on the current distribution of incidents insufficient. Incorporating climate information into public health surveillance systems may help anticipate emerging risk areas and support more effective prevention strategies.

Among the animal groups evaluated, scorpion envenomation showed the largest projected increase under the + 4 °C scenario, indicating greater sensitivity to increasing temperatures. This pattern may be related to the expansion of scorpionism observed over recent decades and to the ecological characteristics of species such as *Tityus serrulatus*, including synanthropic behavior, broad environmental tolerance, and parthenogenetic reproduction, all of which favor rapid population expansion and the colonization of new areas when environmental conditions are favorable (Gracielle & Adalberto, 2021; Lourenço 2022; Guerra-Duarte et al. 2023). The association between higher temperatures and an increased risk of scorpion envenomation has already been reported in São Paulo State (Chiaravalloti-Neto et al. 2023), suggesting that the projected increase under warming scenarios may reflect both physiological responses of scorpions and the expansion of environmentally suitable conditions for their occurrence. Recent evidence further suggests that different species respond differently to climate change, with potential expansions, contractions, or stability in environmentally suitable areas, reinforcing that responses to warming depend on the ecological characteristics of each species (Moura et al. 2025).

Another important finding was the spatial heterogeneity in the response to increasing temperatures. Although the overall trend was an increase in predicted incidence rates, the magnitude of the projected increase varied considerably among municipalities. The observed correlations suggest that landscape characteristics influence how municipalities respond to warming. This spatial heterogeneity is consistent with the multifactorial nature of venomous animal incidents, which result from interactions among climatic conditions, environmental characteristics, and human activities (Martín et al., 2021). Previous studies have shown that factors such as temperature, forest cover, vegetation loss, urbanization, and species richness jointly determine the risk of venomous animal incidents (Schneider et al., 2021; da Silva Freitas et al. 2025). In this context, municipalities with a greater proportion of agricultural land may be more sensitive to warming, whereas more heavily forested areas may respond differently because of their microclimatic conditions and greater environmental stability.

The occurrence of negative projected incidence rate changes in some municipalities further demonstrates that the response to warming is not uniform across the study area. Ectothermic organisms often exhibit nonlinear responses to temperature, characterized by thermal performance curves in which activity, growth, and other physiological processes increase up to an optimal temperature and decline once this threshold is exceeded (Sinclair et al. 2016; Stocker et al. 2024). Consequently, additional increases in temperature may not result in proportional increases in venomous animal incidents and, under certain environmental conditions, may even reduce environmental suitability for some species. These findings suggest that increasing temperatures are likely not only to increase the overall risk of venomous animal incidents but also to redistribute that risk spatially, creating municipalities that may become more vulnerable while others become comparatively less sensitive to warming.

Several limitations should be considered when interpreting these findings. First, the random partitioning of the dataset into training and testing sets did not explicitly account for the temporal and spatial structure of the observations, which may have resulted in slightly optimistic estimates of predictive performance due to the spatial and temporal autocorrelation inherent in the data. In addition, the models did not explicitly incorporate spatiotemporal trends, socioeconomic factors, or differences in local public health surveillance capacity, all of which may influence both the occurrence and reporting of venomous animal incidents. Finally, SINAN records remain subject to underreporting and differences in data completeness among municipalities.

Finally, the simulated scenarios considered changes in temperature only, while all other environmental variables were held constant. Under the + 4 °C scenario, simulated temperatures slightly exceeded the range observed during model training. Therefore, given the limited ability of tree-based algorithms to extrapolate relationships beyond the range of the training data, these results should be interpreted as estimates of the model response to hypothetical warming scenarios rather than quantitative projections of future climatic conditions.

Despite these limitations, this study demonstrates that integrating meteorological and land use and land cover variables can help identify areas that are potentially more vulnerable to the effects of increasing temperatures on venomous animal incidents. Furthermore, the findings show that the factors determining the current spatial distribution of these incidents are not necessarily the same as those governing their response to warming, highlighting the importance of incorporating meteorological information into studies aimed at projecting future risks. Integrating such information into public health surveillance systems may support prevention strategies, planning, and resource allocation. Future studies incorporating comprehensive climate projections, land use and land cover change, spatiotemporal components, and socioeconomic indicators may further improve the predictive performance of these models and enhance their application in public health decision-making.

## Conclusion

This study demonstrated that Random Forest outperformed multiple linear regression, Generalized Additive Models (GAM), and Extreme Gradient Boosting (XGBoost) in predicting scorpion, snake, and spider envenomation across São Paulo State. While land use and land cover variables were the main determinants of the current spatial distribution of incidents, temperature was the factor most strongly associated with the projected changes under warming scenarios. These findings indicate that the environmental drivers of current risk are not necessarily the same as those determining future vulnerability to increasing temperatures. Integrating meteorological and land use and land cover information into predictive models may improve the identification of emerging risk areas and support public health surveillance, prevention strategies, and resource allocation under a changing climate.

## Supplementary Information

Below is the link to the electronic supplementary material.

Supplementary Material 1

## Data Availability

This study analysed secondary data obtained from publicly available sources. The data sources are described in the Methods section.
