# Supplementary material for: Comparison of predictive models for estimating scorpion, snake, and spider envenomation under warming scenarios in São Paulo State, Brazil
Source: Int J Biometeorol. 2026 Nov 3;70(11):294. doi: 10.1007/s00484-026-03353-8 (PMC13731446; doi:10.1007/s00484-026-03353-8)
Supplement: Supplementary file 1 [file 484_2026_3353_MOESM1_ESM.docx]

Supplementary Table 1. Reclassification of MapBiomas land use and land cover classes into the analytical categories used in this study.

| Analytical category | MapBiomas class | Original class name |
| --- | --- | --- |
| Forest | 3 | Forest formation |
|  | 4 | Savanna formation |
|  | 5 | Mangrove |
|  | 6 | Floodable forest |
|  | 49 | Wooded restinga |
| Wetlands and grasslands | 11 | Wetland |
|  | 12 | Grassland |
|  | 29 | Rocky outcrop |
|  | 32 | Salt flat |
|  | 50 | Herbaceous restinga |
| Farming | 9 | Forest plantation |
|  | 15 | Pasture |
|  | 20 | Sugar cane |
|  | 21 | Mosaic of uses |
|  | 39 | Soybean |
|  | 40 | Rice |
|  | 41 | Other temporary crops |
|  | 46 | Coffee |
|  | 47 | Citrus |
|  | 48 | Other perennial crops |
| Urban area | 24 | Urban area |
| Water | 31 | Aquaculture |
|  | 33 | River, lake and ocean |

Supplementary Table 2: Top 20 municipalities with the largest projected increases in incidence rates under the +4°C warming scenario.

| Animal | Rank | IBGE code | Municipality | Mean projected chang | Mean predicted incidence rate (current) | Mean predicted incidence rate (+4°C) |
| --- | --- | --- | --- | --- | --- | --- |
| Scorpions | 1 | 355290 | Taciba | 27.98 | 29.15 | 57.13 |
|  | 2 | 352540 | Jeriquara | 26.13 | 34.61 | 60.74 |
|  | 3 | 352790 | Lutécia | 25.98 | 46.54 | 72.53 |
|  | 4 | 354950 | São José da Bela Vista | 25.75 | 26.24 | 51.99 |
|  | 5 | 354220 | Rancharia | 23.73 | 23.5 | 47.24 |
|  | 6 | 353190 | Morro Agudo | 23.06 | 27.27 | 50.33 |
|  | 7 | 351810 | Guarantã | 22.9 | 35.85 | 58.76 |
|  | 8 | 352920 | Martinópolis | 22.48 | 22.28 | 44.76 |
|  | 9 | 355380 | Taquarituba | 21.53 | 8.9 | 30.42 |
|  | 10 | 352560 | João Ramalho | 21.12 | 37.47 | 58.59 |
|  | 11 | 352420 | Jaborandi | 20.74 | 30.73 | 51.47 |
|  | 12 | 351700 | Getulina | 20.43 | 42.21 | 62.64 |
|  | 13 | 354170 | Quatá | 20.24 | 27.83 | 48.07 |
|  | 14 | 355520 | Turiúba | 20.02 | 83.31 | 103.32 |
|  | 15 | 354350 | Riversul | 19.92 | 33.99 | 53.91 |
|  | 16 | 350980 | Campos Novos Paulista | 19.28 | 36.88 | 56.16 |
|  | 17 | 352880 | Maracaí | 18.99 | 28.54 | 47.53 |
|  | 18 | 355440 | Terra Roxa | 18.9 | 26.31 | 45.21 |
|  | 19 | 351790 | Guaraci | 18.85 | 22.74 | 41.59 |
|  | 20 | 352280 | Itaporanga | 18.74 | 12.96 | 31.69 |
| Snakes | 1 | 350770 | Braúna | 10.49 | 21.64 | 32.13 |
|  | 2 | 351900 | Herculândia | 9.86 | 16.06 | 25.92 |
|  | 3 | 353490 | Pacaembu | 7.16 | 10.62 | 17.79 |
|  | 4 | 351690 | General Salgado | 6.97 | 12.05 | 19.02 |
|  | 5 | 353520 | Palmeira d'Oeste | 6.82 | 15.53 | 22.34 |
|  | 6 | 351600 | Flórida Paulista | 6.81 | 12.24 | 19.05 |
|  | 7 | 351810 | Guarantã | 6.7 | 18.67 | 25.37 |
|  | 8 | 355390 | Tarabai | 6.65 | 16.99 | 23.64 |
|  | 9 | 353190 | Morro Agudo | 6.44 | 7.3 | 13.74 |
|  | 10 | 350420 | Auriflama | 6.39 | 9.17 | 15.56 |
|  | 11 | 354770 | Santo Anastácio | 6.22 | 9.36 | 15.58 |
|  | 12 | 352130 | Ipuã | 5.89 | 11.32 | 17.21 |
|  | 13 | 352650 | Lavínia | 5.64 | 15.69 | 21.33 |
|  | 14 | 352160 | Irapuru | 5.34 | 14.02 | 19.36 |
|  | 15 | 353260 | Nhandeara | 5.32 | 12.2 | 17.53 |
|  | 16 | 351920 | Iacri | 5.25 | 18.92 | 24.17 |
|  | 17 | 350080 | Alfredo Marcondes | 5.18 | 23.73 | 28.91 |
|  | 18 | 352600 | Junqueirópolis | 5.08 | 8.07 | 13.14 |
|  | 19 | 354000 | Pompéia | 5.07 | 8.54 | 13.61 |
|  | 20 | 354765 | Santa Salete | 4.99 | 39.89 | 44.87 |
| Spiders | 1 | 351810 | Guarantã | 8 | 22.8 | 30.8 |
|  | 2 | 354290 | Ribeirão Bonito | 6.7 | 11.84 | 18.54 |
|  | 3 | 353000 | Mira Estrela | 6.66 | 35.12 | 41.78 |
|  | 4 | 352630 | Lagoinha | 6.49 | 40.26 | 46.75 |
|  | 5 | 354790 | Santo Antônio da Alegria | 6.31 | 21.38 | 27.69 |
|  | 6 | 350770 | Braúna | 6.16 | 22.32 | 28.48 |
|  | 7 | 350690 | Bofete | 6.15 | 20.04 | 26.19 |
|  | 8 | 354110 | Presidente Alves | 5.69 | 24.43 | 30.12 |
|  | 9 | 351260 | Coronel Macedo | 5.57 | 27.74 | 33.31 |
|  | 10 | 351700 | Getulina | 5.54 | 17.61 | 23.15 |
|  | 11 | 352970 | Miguelópolis | 5.43 | 11.49 | 16.93 |
|  | 12 | 354860 | São Bento do Sapucaí | 5.37 | 18.43 | 23.8 |
|  | 13 | 352650 | Lavínia | 5.25 | 19.49 | 24.74 |
|  | 14 | 354000 | Pompéia | 5.22 | 7.83 | 13.05 |
|  | 15 | 351690 | General Salgado | 5.2 | 12.53 | 17.73 |
|  | 16 | 350020 | Adolfo | 5.19 | 25.75 | 30.95 |
|  | 17 | 355390 | Tarabai | 5.08 | 19.61 | 24.69 |
|  | 18 | 350830 | Cabrália Paulista | 5.04 | 26.08 | 31.12 |
|  | 19 | 353370 | Ocauçu | 5 | 33.21 | 38.22 |
|  | 20 | 351470 | Echaporã | 4.85 | 20.71 | 25.56 |

Supplementary Table 3: Supplementary Table 3. Top 20 municipalities with the largest projected reductions (or smallest increases) in incidence rates under the +4°C warming scenario.

| Animal | Rank | IBGE code | Municipality | Mean projected chang | Mean predicted incidence rate (current) | Mean predicted incidence rate (+4°C) |
| --- | --- | --- | --- | --- | --- | --- |
| Scorpions | 1 | 350720 | Borá | -18.48 | 118.35 | 99.87 |
|  | 2 | 350315 | Arapeí | -16.47 | 71.35 | 54.89 |
|  | 3 | 355530 | Turmalina | -13.59 | 135.13 | 121.54 |
|  | 4 | 353286 | Nova Castilho | -10.34 | 131.69 | 121.35 |
|  | 5 | 355475 | Trabiju | -8.53 | 81.84 | 73.32 |
|  | 6 | 352910 | Marinópolis | -8.21 | 93.98 | 85.76 |
|  | 7 | 352585 | Jumirim | -8.01 | 57.32 | 49.31 |
|  | 8 | 350260 | Aparecida d'Oeste | -5.41 | 106.28 | 100.87 |
|  | 9 | 351565 | Fernão | -4.68 | 82.03 | 77.35 |
|  | 10 | 350995 | Canas | -4.07 | 29.57 | 25.5 |
|  | 11 | 352070 | Indiaporã | -4.01 | 103.91 | 99.91 |
|  | 12 | 352120 | Iporanga | -3.64 | 23.45 | 19.81 |
|  | 13 | 352700 | Lindóia | -3.33 | 28.24 | 24.91 |
|  | 14 | 355570 | União Paulista | -2.58 | 79.45 | 76.87 |
|  | 15 | 354380 | Rinópolis | -2.3 | 77.82 | 75.51 |
|  | 16 | 355465 | Torre de Pedra | -2.23 | 39.99 | 37.76 |
|  | 17 | 354280 | Ribeira | -2.08 | 27.79 | 25.71 |
|  | 18 | 353625 | Parisi | -2.07 | 76.28 | 74.21 |
|  | 19 | 350745 | Borebi | -1.81 | 56.98 | 55.17 |
|  | 20 | 352215 | Itaoca | -1.74 | 29.13 | 27.39 |
| Snakes | 1 | 350720 | Borá | -13.21 | 70.24 | 57.03 |
|  | 2 | 355475 | Trabiju | -6.03 | 66.21 | 60.19 |
|  | 3 | 355460 | Timburi | -5.79 | 46.57 | 40.78 |
|  | 4 | 350995 | Canas | -5.26 | 15.51 | 10.24 |
|  | 5 | 350500 | Barão de Antonina | -4.72 | 36.92 | 32.2 |
|  | 6 | 354625 | Santa Cruz da Esperança | -4.59 | 31.08 | 26.48 |
|  | 7 | 352585 | Jumirim | -4.39 | 24.34 | 19.95 |
|  | 8 | 353380 | Óleo | -4.1 | 43.91 | 39.81 |
|  | 9 | 351090 | Cássia dos Coqueiros | -4.09 | 44.69 | 40.6 |
|  | 10 | 354830 | Santo Expedito | -3.88 | 47.91 | 44.03 |
|  | 11 | 355530 | Turmalina | -3.17 | 42.32 | 39.16 |
|  | 12 | 354620 | Santa Cruz da Conceição | -3.06 | 19.96 | 16.9 |
|  | 13 | 355465 | Torre de Pedra | -2.72 | 26.21 | 23.49 |
|  | 14 | 355590 | Uru | -2.56 | 67.16 | 64.6 |
|  | 15 | 354960 | São José do Barreiro | -2.53 | 36.44 | 33.91 |
|  | 16 | 351015 | Canitar | -2.52 | 13.56 | 11.05 |
|  | 17 | 355520 | Turiúba | -2.4 | 53.05 | 50.65 |
|  | 18 | 350315 | Arapeí | -2.38 | 43.61 | 41.23 |
|  | 19 | 354165 | Quadra | -2.35 | 21.44 | 19.09 |
|  | 20 | 353286 | Nova Castilho | -2.21 | 75.31 | 73.1 |
| Spiders | 1 | 350720 | Borá | -9.1 | 96.81 | 87.71 |
|  | 2 | 350995 | Canas | -3.78 | 17.83 | 14.05 |
|  | 3 | 354165 | Quadra | -2.68 | 28.15 | 25.48 |
|  | 4 | 353286 | Nova Castilho | -2.45 | 64.12 | 61.68 |
|  | 5 | 350730 | Boracéia | -2.05 | 21.58 | 19.53 |
|  | 6 | 354620 | Santa Cruz da Conceição | -1.81 | 28.24 | 26.44 |
|  | 7 | 355465 | Torre de Pedra | -1.79 | 49.14 | 47.35 |
|  | 8 | 351015 | Canitar | -1.78 | 9.87 | 8.09 |
|  | 9 | 352700 | Lindóia | -1.66 | 19.27 | 17.6 |
|  | 10 | 354030 | Pontes Gestal | -1.49 | 32.85 | 31.36 |
|  | 11 | 350360 | Areiópolis | -1.28 | 8.4 | 7.12 |
|  | 12 | 351565 | Fernão | -1.14 | 56.39 | 55.24 |
|  | 13 | 353170 | Monteiro Lobato | -1.12 | 47.38 | 46.26 |
|  | 14 | 350075 | Alambari | -1.11 | 16.83 | 15.72 |
|  | 15 | 353610 | Pardinho | -1.08 | 24.2 | 23.12 |
|  | 16 | 353715 | Pedrinhas Paulista | -0.95 | 28.86 | 27.91 |
|  | 17 | 352200 | Itaju | -0.9 | 26.31 | 25.41 |
|  | 18 | 350350 | Areias | -0.77 | 31.54 | 30.77 |
|  | 19 | 354280 | Ribeira | -0.76 | 29.57 | 28.81 |
|  | 20 | 350335 | Arco-Íris | -0.73 | 46 | 45.27 |
